# Beneficial Effects of ACC Deaminase-Producing Rhizobacteria on the Drought Stress Resistance of *Coffea arabica* L.

**DOI:** 10.3390/plants14071084

**Published:** 2025-04-01

**Authors:** Yesenia Jasso-Arreola, J. Antonio Ibarra, Flor de Fátima Rosas-Cárdenas, Paulina Estrada-de los Santos

**Affiliations:** 1 Instituto Politécnico Nacional, Escuela Nacional de Ciencias Biológicas, Prol. Carpio y Plan de Ayala s/n, Col. Santo Tomás, Alcaldía Miguel Hidalgo, Ciudad de México 11340, Mexico; yjassoa@ipn.mx (Y.J.-A.); jaig19@gmail.com (J.A.I.); 2 Instituto Politécnico Nacional, Centro de Estudios Científicos y Tecnológicos 16 “Hidalgo”, Carretera Pachuca-Actopan km 1+500, San Agustín Tlaxiaca 42162, Mexico; 3 Instituto Politécnico Nacional, Centro de Investigaciónen Biotecnología Aplicada, Ex-Hacienda SanJuan Molino Carretera Estatal Tecuexcomac-Tepetitla Km 1.5, Tepetitla 90700, Mexico; frosasc@ipn.mx

**Keywords:** drought resistance, stress acclimation, growth promotion, rhizobacterial isolation, *Pantoea*

## Abstract

Given the challenges of climate change, effective adaptation strategies for crops like coffee are crucial. This study evaluated twelve 1-aminocyclopropane-1-carboxylate deaminase-producing bacterial strains selectively isolated from the rhizosphere of *Coffea arabica* L. cv. Costa Rica 95 in a plantation located in Veracruz, Mexico, focusing on their potential to enhance drought resistance. The strains, representing seven genera from the Gamma-proteobacteria and Bacteroidota groups, were characterized for growth-promoting traits, including ACC deaminase activity, indole-3-acetic acid (IAA) synthesis, phosphates solubilization, siderophore production, and nitrogen fixation. Strains of the genus *Pantoea* exhibited higher ACC deaminase activity, phosphate solubilization, and IAA synthesis, while others, such as *Sphingobacterium* and *Chryseobacterium*, showed limited plant growth-promoting traits. A pot experiment was conducted with coffee plants subjected to either full irrigation (soil with 85% volumetric water content) or drought (soil with 55% volumetric water content) conditions, along with inoculation with the isolated strains. Plants inoculated with *Pantoea* sp. RCa62 demonstrated improved growth metrics and physiological traits under drought, including higher leaf area, relative water content (RWC), biomass, and root development compared to uninoculated controls. Similar results were observed with *Serratia* sp. RCa28 and *Pantoea* sp. RCa31 under full irrigation conditions. *Pantoea* sp. RCa62 exhibited superior root development under stress, contributing to overall plant development. Proline accumulation was significantly higher in drought-stressed, non-inoculated plants compared to those inoculated with *Pantoea* sp. RCa62. This research highlights the potential of *Pantoea* sp. RCa62 to enhance coffee plant resilience to drought and underscores the need for field application and further validation of these bioinoculants in sustainable agricultural practices.

## 1. Introduction

Coffee is a plant in the genus *Coffea* that belongs to the Rubiaceae family. While many wild species exist, the most commercially significant are *C. arabica* L. and *C. canephora* (Pierre) ex A. Froehner. *C. arabica*, which originated in southern Ethiopia [1], is globally famous for its aroma, sweetness, and variations in acidity and flavor. Coffee is one of the most traded commodities globally [2]. This plant is cultivated in over 70 countries by 25 million farmers, most of them smallholders in developing countries [3]. Furthermore, agroforestry systems, where most coffee is cultivated in many coffee-producing areas, serve as important reservoirs of cultural richness, biodiversity, and ecological processes [4,5,6].

The phenology and, consequently, the yield and quality of *C. arabica* are strongly dependent on climatic conditions [7]. Prior studies have reported changes in the production and accumulation of several metabolites along with adverse impacts of drought on coffee leaf relative water content (RWC), stomatal conductance, leaf water potential, net carbon assimilation rate, transpiration rate, and, as a result, growth and yield [8,9,10,11]. Hence, climate change presents a significant challenge to the cultivation of coffee in various regions, with considerable reductions anticipated in suitable areas for cultivation [12,13,14,15]. Given this crop’s social, economic, and environmental importance, the potential impact of climate change on coffee production is alarming and requires adaptation and mitigation strategies [16].

A significant limitation of agricultural production in climate change is drought [17], as the lack of water affects plant physiology, development, and crop yield and quality [18,19,20]. The search for new fertilization alternatives that allow production, even under these stressful conditions, becomes more critical. One of these possible strategies is to use microorganisms as biofertilizers, enhancing plant acclimatization, improving soil structure and fertility, and increasing crop yield. Specifically, the beneficial organisms that inhabit the rhizosphere, commonly referred to as plant growth-promoting rhizobacteria (PGPR), possess the capability to preserve or enhance crop growth and production, even in the presence of drought-related environmental stressors, and do not necessarily have to establish endosymbiotic relations with the plant to confer beneficial effects [21,22].

PGPR can improve crop performance through the bacterial synthesis of phytohormones [23]; the increase of nutrient availability through nitrogen fixation, phosphate solubilization, or siderophore production; and the secretion of protective substances such as volatile organic compounds and extracellular polymeric substances [24]. As a result, the interaction with these beneficial microorganisms favors the modulation of the expression profile of several plant genes, either those directly involved in protecting cells from stress (such as enzymes related to osmolyte production or antioxidant activity) or those including regulatory proteins that modulate gene expression (such as transcription factors) [25,26]. In consequence, plants associated with PGPR exhibit, for instance, the regulation of plant hormonal levels, the activation of antioxidant systems along with the consequent reduction in the concentration of malondialdehyde due to damage to cell membranes, and the regulation of plant metabolite synthesis. All these mechanisms contribute to increased growth and better development of the plants [23,24,27]. Among PGPR, those capable of producing ACC deaminase (ACCd-PGPR) are of particular interest, as they degrade the precursor of ethylene, 1-aminocyclopropane-1-carboxylic acid (ACC), into α-ketobutyrate and ammonia. High levels of ethylene over long periods can harm plant health, growth, and productivity [28,29,30]. Accordingly, many studies have documented that inoculation with ACCd-PGPR helps to enhance drought resistance in a wide variety of plants [31,32,33,34,35,36].

To date, insufficient data is available regarding the isolation, characterization, and identification of PGPR from the rhizosphere of coffee plants [37]; even more, no studies explicitly focus on the exploration of ACCd-PGPR. Further, it is essential to consider that, beyond the plant growth-promoting characteristics described for the strains, a significant amount of research has been conducted on seeds and sterilized substrate or soil, reporting positive effects with PGPR inoculation under abiotic stress conditions [38,39,40,41,42,43], still overlooking the bacterial ability to confer tolerance to stressors under conditions that are closer to those found in a productive context. Several strains can promote plant growth and confer resistance to environmental stressors when inoculated onto sterile seeds and planted in sterile soil or substrate. They may also exhibit promising plant growth-promoting characteristics when tested in vitro as axenic cultures. However, the effects of these microorganisms may be less pronounced when the same strains are examined in non-sterile substrates or on older plants [44,45,46]. This is because microorganisms inoculated under sterile conditions do not encounter competitors that might hinder their establishment. Additionally, environmental conditions directly affect the physiological state of plants, which subsequently affects the composition of root exudates and modifies the potential for plant-bacteria interactions [47]. Since root colonization is a multi-step process that depends on species-specific chemical interactions [48], searching for PGPR naturally associated with the crops of interest becomes relevant.

Hence, in this study, we aimed to isolate ACCd-producing rhizobacteria from the rhizosphere of *C. arabica* cv. Costa Rica 95 in a central Veracruz coffee plantation and determine the impact of these strains on drought resistance strategies on young *C. arabica* plants exposed to moderate and prolonged drought. This study addresses the knowledge gap regarding ACC deaminase-producing bacteria associated with coffee plants in productive plantations. Even more, we have not found other studies directly assessing the effect of potential plant growth-promoting strains on coffee drought resistance under conditions that more closely resemble real-world agricultural settings (inoculation in young plants and non-sterile soil). This approach offers new insights into the practical application of these bacteria for enhancing coffee plant resilience in actual production environments increasingly threatened by climate change.

## 2. Results

### 2.1. Identification and Characterization of ACCd-Producing Rhizobacteria Strains

Twelve strains from seven genera within the bacterial groups Gamma-proteobacteria and Bacteroidota were isolated from the coffee rhizosphere (Table 1; Figure S1). The growth-promoting traits present in these microorganisms vary significantly (Table 1; Figure S2), allowing the strains to form three clusters. The three strains of the genus *Pantoea* were characterized by moderate to high relative ACCd activity, high PSI, and increased IAA production. In contrast, the group consisting of *Sphingobacterium* sp. RCa08 and RCa20, *Stenotrophomonas* sp. RCa07, and *Chryseobacterium* sp. RCa13 exhibited moderate to low ACCd activity; no other plant growth-promoting characteristics were found. Finally, the third cluster includes *Pseudomonas* sp. RCa18 and RCa58, *Raoultella* sp. RCa12, and *Serratia* sp. RCa01 and RCa28. This cluster showed varying ACCd activity from low to very high, high iron solubilization by siderophores, moderate PSI, and marginal IAA production, particularly by *Serratia* sp. RCa01. None of the strains fixed atmospheric nitrogen or produced IAA through a tryptophan-independent pathway.

All the strains exhibited ACCd activity; *Pseudomonas* sp. RCa18 and *Pantoea* sp. RCa31 and RCa62 show the highest ACCd activity. Among the twelve strains evaluated, five were found to produce siderophores. *Serratia* sp. RCa01 is the isolate with the largest solubilization halo after eight days of incubation, compared to *Pseudomonas* sp. RCa58. On the other hand, eight strains could solubilize phosphates, with the genus *Pantoea* exhibiting higher PSI than the other genera. Regarding IAA production, five strains were found to produce IAA through a tryptophan-dependent pathway. Notably, the three strains of the genus *Pantoea* exhibited significant IAA production, with *Pantoea* sp. RCa62 showing the highest capacity (Table 1). It is important to mention that this characteristic could not be evaluated in strains RCa07 and RCa13, as they did not grow in the Jain and Patriquin medium.

### 2.2. Effect of ACC Deaminase-Producing Strains on the Growth and Physiological Status of Coffea arabica L.

#### 2.2.1. Differentiation Between Treatments

**Figure 1:**
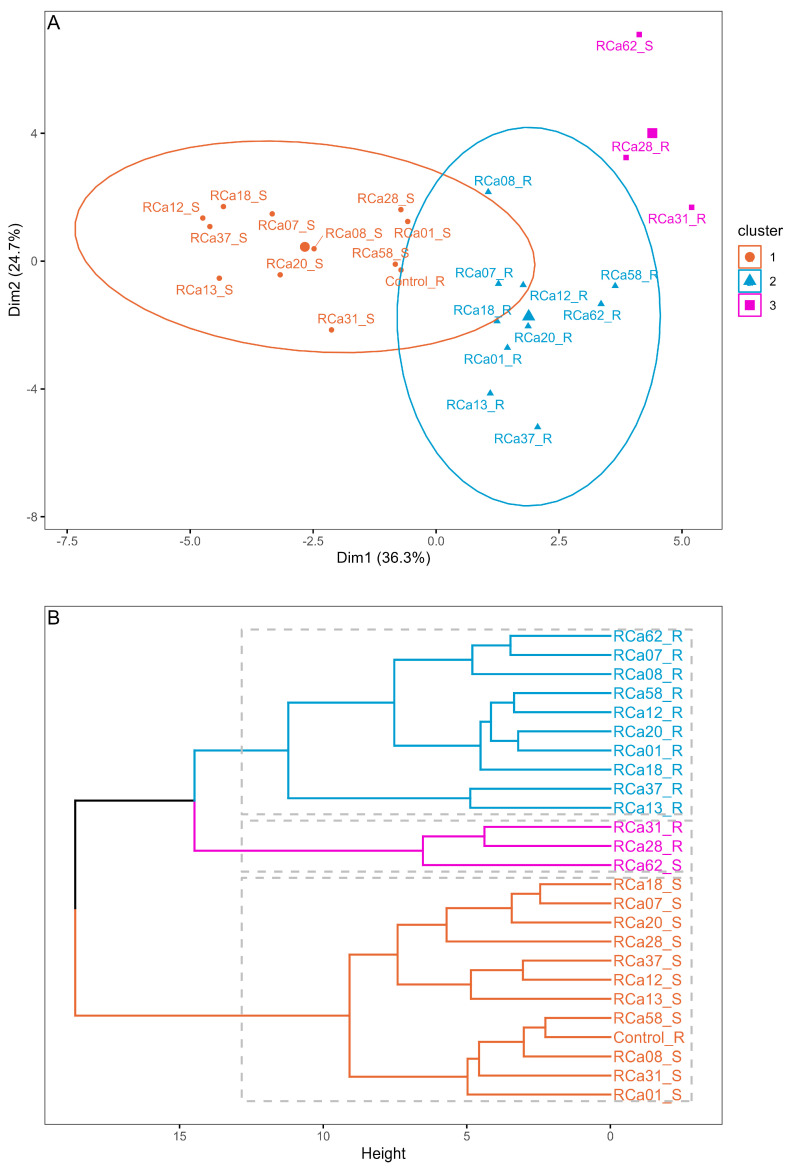
Clustering (**A**) and dendrogram (**B**) of plants under irrigation and inoculation treatments. The cluster calculations were performed using the k-means method. Plants under regular irrigation (85% RWC) are marked with the letter R, while those under drought (55% RWC) are marked with the letter S.

**Figure 2:**
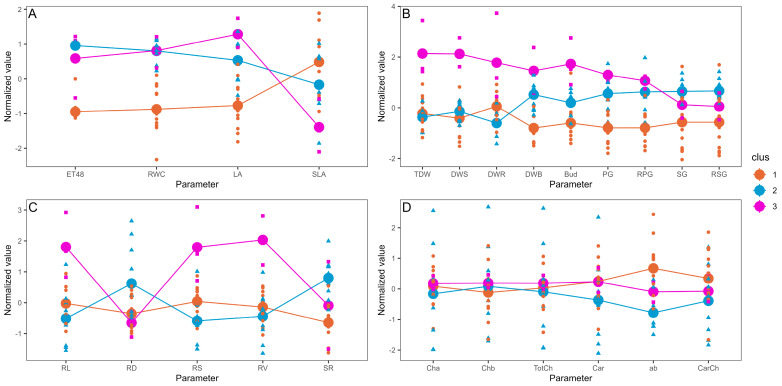
Normalized mean values for each cluster. The thick points represent the mean normalized value for each parameter. The symbols represent the individual normalized data for each element of the cluster. Cluster calculations were performed using the k-means method. (**A**) Effects on evapotranspiration and leaf functional attributes. (**B**) Effects on plant growth and development. (**C**) Effects on root development. (**D**) Effects of pigment accumulation. ET48, evapotranspiration over 48 h; LA, leaf area; RWC, relative water content; SLA, specific leaf area; PG, primary growth; RPG, relative primary growth; SG, secondary growth; RSG, relative secondary growth; DWS, dry weight of the shoot; DWR, dry weight of the root; TDW, total dry weight; Bud, number of leaf buds; FWB, fresh weight of leaf buds; DWB, dry weight of leaf buds; RL, total root length; RD, root diameter; RS, total root surface; RV, total root volume; SR, shoot-to-root ratio; Cha, chlorophyll a concentration; Chb, chlorophyll b concentration; TotCh, total chlorophyll concentration; Car, carotenoids concentration; ab, chlorophyll a/b ratio; CarCh, carotenoids/chlorophyll ratio.

Based on the pot experiment results, cluster analysis revealed a clear separation among most of the plants under water scarcity (S, 55% VWC, cluster 1), the majority of well-irrigated plants (R, 85% VWC, cluster 2), and plants under full irrigation inoculated with *Serratia* sp. RCa28 and *Pantoea* sp. RCa31, as well as drought-stressed plants inoculated with *Pantoea* sp. RCa62 (cluster 3) (Figure 1). Figure 2 indicates that cluster 3 displayed higher values in leaf area, primary growth, biomass, leaf bud production, and root development. Notably, the cluster with the highest shoot-to-root ratio includes most plants with regular irrigation, whereas plants in cluster 3 have an intermediate shoot-to-root ratio. PCA, in which this cluster analysis was performed, explains 63.1% of the variability. Mean values for each irrigation treatment can be observed in Table S1, again showing a clear differentiation between growth and functional attributes in plants under irrigation and drought.

#### 2.2.2. Effects on Evapotranspiration

**Figure 3:**
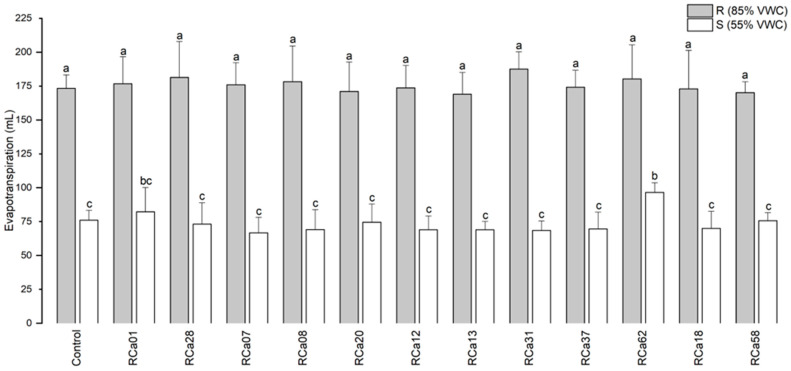
Mean evapotranspiration of *C. arabica* L. cv. Costa Rica 95 over 48 h. Plot represents mean ± CI 95% (n = 7) for the plants irrigated at 85% VWC (R) and at 55% VWC (S). Different letters indicate significant differences between groups (*p* < 0.05; post-hoc Duncan).

A notable decrease in evapotranspiration was observed in plants exposed to drought conditions (S, 55% VWC). However, in plants inoculated with *Pantoea* sp. RCa62, this parameter was higher than most of the other drought-stressed groups, except for *Serratia* sp. RCa01 (Figure 3).

#### 2.2.3. Effects on Leaf Functional Traits

**Figure 4:**
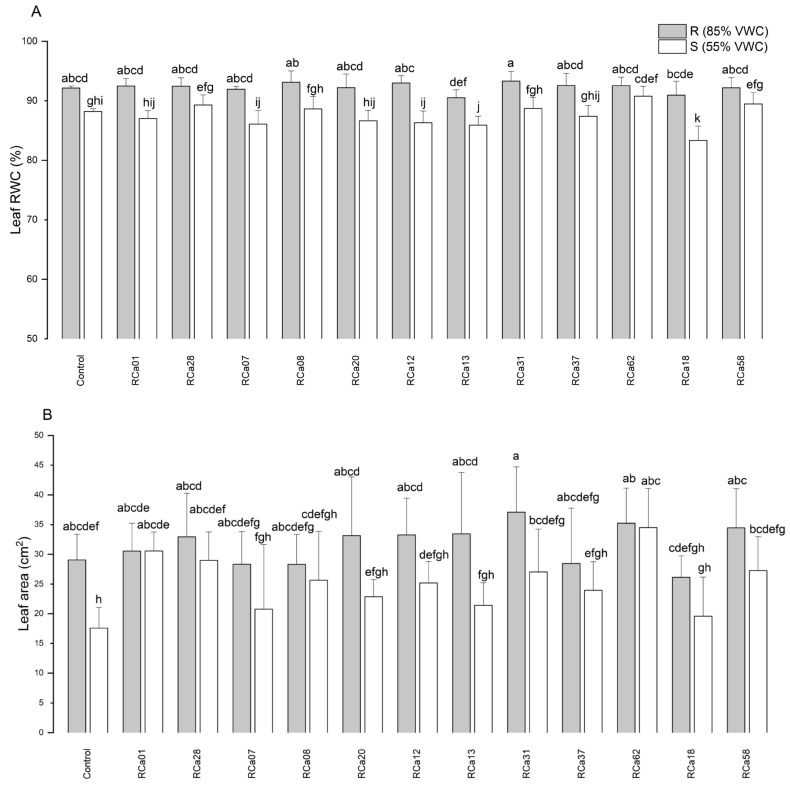
Leaf relative water content (RWC) (**A**) and leaf area (**B**) of *C. arabica* L. cv. Costa Rica 95. Plot represents mean ± CI 95% (n = 7) for the plants irrigated at 85% VWC (R) and at 55% VWC (S). Different letters indicate significant differences between groups (*p* < 0.05; post-hoc Duncan).

**Figure 5:**
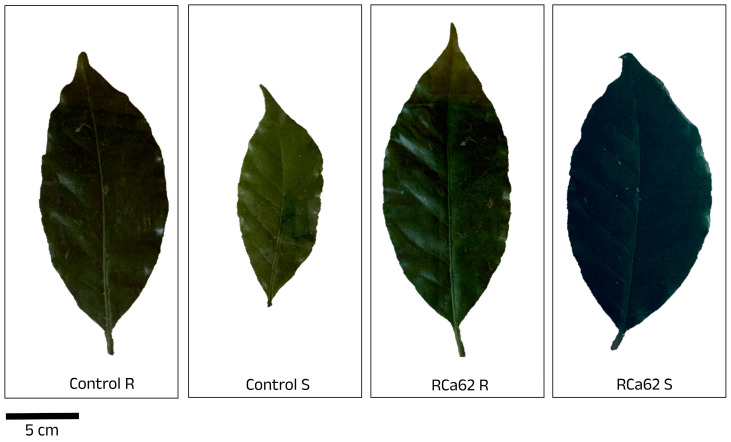
Leaf area of uninoculated (Control) and inoculated plants with *Pantoea* sp. RCa62 under full irrigation (R) and drought stress (S) treatments.

The impact of the treatments on leaf relative water content (RWC), leaf area (Figure 4), and specific leaf area (SLA) was analyzed. The R group showed significantly higher values for RWC. Among these, those associated with *Pantoea* sp. RCa31 exhibited the highest RWC, markedly surpassing plants treated with *Chryseobacterium* sp. RCa13 and *Pseudomonas* sp. RCa18. All R groups were statistically similar to uninoculated controls. Likewise, drought-stressed plants inoculated with *Chryseobacterium* sp. RCa13 and *Pseudomonas* sp. RCa18 had the lowest RWC, even less than uninoculated controls. Notably, plants associated with *Pantoea* sp. RCa62 maintained higher RWC levels than drought-stressed uninoculated individuals and was comparable to many well-watered plants. Regarding leaf area, plants inoculated with *Pantoea* sp. RCa31, under optimal irrigation conditions, had the largest leaves, which were significantly different from those treated with *Pseudomonas* sp. RCa18 under similar conditions. The smallest leaves were observed in non-inoculated plants under drought stress (Figure 5). On the other hand, *Pantoea* sp. RCa62, *Serratia* sp. RCa01, RCa28, *Pantoea* sp. RCa31, and *Pseudomonas* sp. RCa58 exhibited positive effects under drought, with leaf areas greater than those of uninoculated controls. Specifically, the leaf areas in RCa01, RCa28, and RCa62 were comparable to those of non-stressed plants across all inoculation treatments. No significant differences in SLA were found among the groups.

#### 2.2.4. Effect on Plant Growth

The effects of irrigation and inoculation treatments on many parameters, including growth and weight, were analyzed (Supplementary Table S1). These parameters in stressed plants inoculated with *Pantoea* sp. RCa62 were notably higher than those exhibited by most of the groups under water deficit and even comparable to those of the groups without this stressor.

Absolute and relative primary growth (Table 2) in plants under adequate water conditions inoculated with *Pseudomonas* sp. RCa58, RCa18, and *Serratia* sp. RCa28 were elevated but had no significant differences compared to uninoculated well-watered controls.

The poorest performance was observed on plants under drought conditions inoculated with *Pantoea* sp. RCa37, although they did not differ from uninoculated plants. It is important to note that the initial length of the plants was homogeneous between all treatments (ANOVA, *p* > 0.05). In the same way, there is no correlation between the initial plant height and the absolute primary growth (Pearson test, r^2^ = 0.0128, *p* > 0.05) and relative primary growth (Pearson test, r^2^ = 0.0105, *p* > 0.05). Regarding secondary growth, none of the inoculation treatments differed from the uninoculated controls for each watering group; however, mean secondary growth rates showed that stressed plants inoculated with *Stenotrophomonas* RCa07, *Sphingobacterium* sp. RCa20, *Raoultella* sp. RCa12, *Chryseobacterium* sp. RCa13, *Pseudomonas* sp. RCa18, and *Pantoea* sp. RCa37 had equal or even thinner stems after two months of water stress. The effect of irrigation treatments is evident on the assessed primary and secondary growth parameters.

Regarding the dry weight of the plants after the inoculation and watering treatments, the root weights of well-irrigated plants were lower than expected compared to the stressed groups (Table 3). It is also interesting that plants inoculated with *Pantoea* sp. RCa62 exhibit the highest value for shoot weight, even when compared to some well-watered plants, including the control group. Furthermore, it was observed that they also had the best root development. No differences in total dry weight were detected by ANOVA (*p* > 0.05).

Concerning leaf bud production, plants under abundant watering conditions are in symbiosis with *Pantoea* sp. RCa31, which stands out, while those with the fewest buds’ production were those subjected to water deficit and inoculated with *Pantoea* sp. RCa37 (Table 4). When analyzing the fresh weight of the sprouts, the effect of *Pantoea* sp. RCa31 is again notable compared to other strains under watering conditions (e.g., *Stenotrophomonas* sp. RCa07, *Sphingobacterium* sp. RCa08, *Chryseobacterium* sp. RCa13, and *Pseudomonas* sp. RCa18); similar trends are observed when exploring the effect on the dry weight of the leaf buds. In the case of drought-stressed plants, the effect of *Pantoea* sp. RCa62 is again evident, showing fresh and dry sprout weights greater than those of the control group and plants in symbiosis with *Stenotrophomonas* sp. RCa07, *Sphingobacterium* sp. RCa08, RCa20, *Raoultella* sp. RCa12, *Pantoea* sp. RCa37, *Pseudomonas* sp. RCa58, and RCa18. *Pantoea* sp. RCa62 provided a bud production comparable to the plant groups under 85% VWC irrigation conditions.

#### 2.2.5. Effects on Root Development

**Figure 6:**
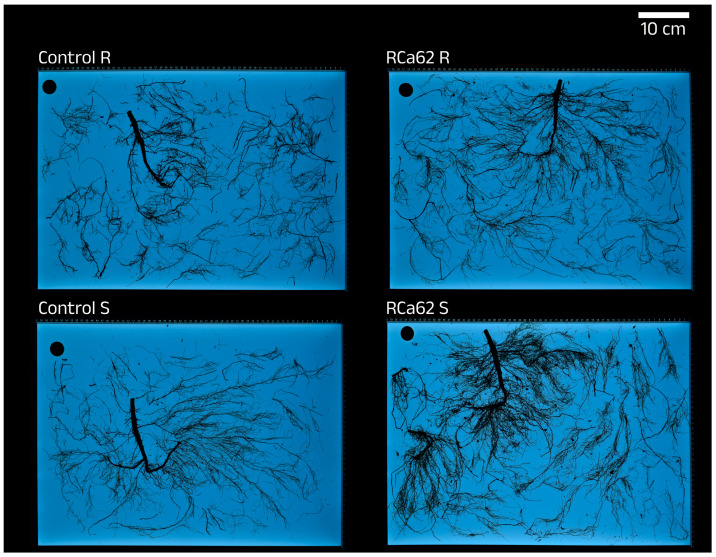
Root architecture of uninoculated (Control) and inoculated plants with *Pantoea* sp. RCa62 under full irrigation (R) and drought stress (S) treatments.

There is a clear difference between root morphology and development between irrigation treatments since stressed plants show longer and thinner roots with wider surfaces (Table 5). In addition, the image analyses indicated that the percentage of tap roots in well-watered plants is significantly higher than in stressed plants (R = 2.39% ± 0.10, S = 1.91% ± 0.06; U Mann-Whitney, *p* ˂ 0.001). In contrast, the latter prioritizes the development of thinner secondary roots. Furthermore, the dry-weight shoot-to-root ratio is notoriously lower in stressed plants. Even though there was no statistical significance in the root morphology parameters, the broadest roots in plants were consistently associated with *Pantoea* sp. RCa62 and were observed under drought conditions (Figure 6). Moreover, it was observed that the groups that exhibited the best performance in the experiment (well-watered and inoculated with *Serratia* sp. RCa28 and *Pantoea* sp. RCa31; subjected to drought and inoculated with *Pantoea* sp. RCa62) had intermediate shoot-to-root ratios compared to the other clusters (Figure 2C).

#### 2.2.6. Effect on Pigment Concentrations

No differences were found in chlorophyll a, b, or total chlorophylls, nor in carotenoid concentration. However, the chlorophyll a/b ratio was higher in stressed plants. Individuals under drought and associated with *Serratia* sp. RCa28 exhibited the highest chlorophyll a/b ratio. A higher carotenoids/chlorophyll ratio can also be observed in drought-stressed plants (Table S2).

#### 2.2.7. Effect on Proline Accumulation

**Figure 7:**
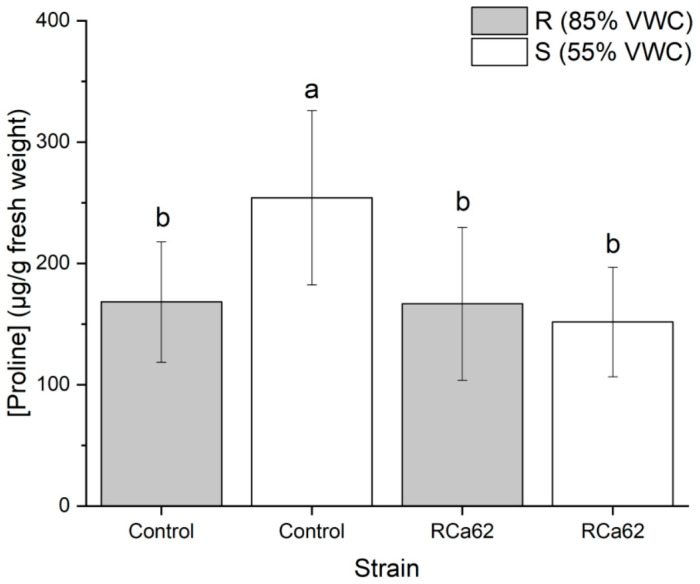
Proline accumulation on *C. arabica* L. cv. Costa Rica 95 leaves. Plot represents mean ± CI 95% (n = 7) for the plants irrigated at 85% VWC (R) and 55% VWC (S). Different letters indicate significant differences between groups (*p* < 0.05; post-hoc Duncan).

When comparing plants inoculated with *Pantoea* sp. RCa62 to the control group, changes in proline production were observed. Individuals not inoculated and exposed to drought conditions showed increased osmolyte accumulation (Figure 7).

## 3. Discussion

### 3.1. ACCd-Producing Rhizobacteria Strains

Climate change conditions make it necessary to seek adaptation strategies for agricultural systems to maintain productivity and improve crop quality, particularly for crops with more potential vulnerabilities, such as coffee [11,14]. ACC deaminase-producing bacteria have attracted considerable attention due to their ability to lower ethylene peaks associated with physiological stress, thereby promoting plant growth. This quality makes them appealing as potential ingredients in bioinoculant formulations [29]. This is the first study exploring ACCd-PGPR in *C. arabica* L. under agricultural conditions. Using a selective medium with ACC as the only nitrogen source, twelve strains were isolated from the rhizospheric soil of *C. arabica* var. Costa Rica 95. The strains belong to seven genera, all of which are Gram-negative, consistent with previous studies that describe the prevalence of such bacteria associated with coffee [49,50]. All the identified genera have been described as ACC deaminase producers and plant growth promoters when inoculated into various plant species under abiotic stress conditions [38,39,40,41,42,43,51,52,53,54]. In all cases, the strains exhibited more significant ACC deaminase activity than the minimum required for regulating tissue ethylene concentration and promoting plant growth, as reported by Penrose and Glick [55].

Microorganisms found in this research, such as *Serratia* and *Pseudomonas,* have been found in the rhizosphere of wild *C. arabica* in the forests of Ethiopia, as well as bacteria of the genus *Erwinia*, a member of the Erwiniaceae family [49], to which the genus *Pantoea* also belongs and shares many phenotypic features [56]. Moreover, some *Erwinia* species have been reclassified as *Pantoea* [57], a genus identified in this study as a member of root bacterial communities. Here, *Pantoea* strains were found displaying traits of ACC deaminase production, IAA, and phosphate solubilization, the latter of which was described by Muleta et al. (2013) [58] for *Erwinia* strains. Similarly, *Stenotrophomonas* has been previously reported as an endophytic genus in coffee roots and other tissues as a potential biocontrol agent [50,59]. On the other hand, *Sphingobacterium* has not been identified as a genus in the coffee rhizosphere; however, it has been found in wastewater from coffee bean processing. Nevertheless, other bacteria from the order Sphingobacteriales have been noted as dominant in the rhizosphere of this crop [60,61].

Regarding the microorganisms isolated in this study, both *Serratia* strains (RCa01 and RCa28), *Raoultella* sp. RCa12, and *Pseudomonas* (RCa18 and RCa58) showed the ability to produce siderophores, previously documented for these genera. For example, in *Serratia*, a siderophore called serratioquelline, a catecholate, has been identified in various strains of the genus [62,63] and is associated with plant growth promotion under abiotic stress [53]. Similarly, the ability to synthesize siderophores has been identified in *Raoultella* [64] and *Pseudomonas*, with the latter also providing antagonistic activity against plant pathogenic fungi [65,66].

The phosphate solubilization capability present in the strains of *Serratia*, *Raoultella*, *Pseudomonas*, and *Pantoea* becomes particularly relevant as this macronutrient is a frequent limiting factor to plant development [67] and tends to be scarce under drought conditions. In dry soil, the size of the water-filled pores decreases significantly, reducing the mobility of this element [24]. In addition, the diminished water mobility from the soil to the plants through the root system also reduces the movement of dissolved nutrients and, hence, impacts plant nutrition [67]. Among these genera, the PSI of *Pantoea* strains stands out compared to the others that exhibited activity. This genus has been widely documented as a phosphate solubilizer [68,69,70].

Also remarkable is the production of IAA by *Pantoea* strains and *Serratia* sp. RCa28, although the latter produces it in smaller amounts. The synthesis of IAA has been reported for both genera [53,71], and it is particularly significant due to the crucial role this phytohormone plays in regulating plant responses to water stress. It is involved in optimal root system development, regulating gene expression related to protection against oxidative damage caused by water scarcity, and interacting with ABA in controlling stomatal opening and closing.

### 3.2. Bacterial Effect on Evapotranspiration

Regarding the bacterial effect on *C. arabica*’s resistance to drought, ANOVA showed a significant effect of irrigation and bacterial inoculation treatments on young *C. arabica* cv. Costa Rica 95 in many of the parameters assessed. In the same way, the differentiation of the clusters indicates an effective establishment of the irrigation and drought treatments, as well as the distinction of strains with greater plant growth-promoting effects under each type of water availability treatment (full irrigation, plants associated with *Serratia* sp. RCa28 and *Pantoea* sp. RCa31; drought stress, plants related to *Pantoea* sp. RCa62).

When assessing water consumption through evapotranspiration, it’s important to note that water movement from the soil to the atmosphere involves two key components: evaporation, which transfers water directly from the soil to the atmosphere, and transpiration, which occurs through the soil-root-shoot-atmosphere continuum [72]. Evapotranspiration is highly sensitive to soil water availability, particularly in the *Coffea* genus. Under drought conditions, coffee plants often exhibit stomatal closure as an early response to prevent excessive water loss through transpiration [73,74,75,76]. This explains the significant differences in evapotranspiration observed between drought-stressed groups and those with adequate water availability. These results are consistent with Avila et al. [77] and Naves [78], who found reductions in the evapotranspiration of potted young plants and adult coffee plants under field conditions subjected to water restrictions employing a system of rain exclusion, respectively. However, when water deficiency persists for prolonged periods, as in this study, the decline in stomatal conductance limits CO_2_ assimilation, ultimately affecting growth and productivity [79,80,81,82,83]. This is especially evident in coffee, which has lower stomatal conductance than other tropical plants [74]. Notably, plants inoculated with *Pantoea* sp. RCa62 displayed increased evapotranspiration despite moderate water restrictions. This is particularly remarkable given that these plants also exhibited a larger leaf area, a key parameter directly linked to photosynthetic surface area and productivity—especially in coffee under prolonged water stress [84].

### 3.3. Bacterial Effect on Leaf Functional Traits

Reducing the leaf area in *C. arabica* under drought conditions is a strategy to evade water stress by minimizing water loss through transpiration [84,85]. This mechanism can be confirmed with the overall means of leaf area of stressed plants compared to fully irrigated plants and becomes especially evident in non-inoculated individuals under drought conditions. In contrast, drought-stressed plants inoculated with *Pantoea* sp. RCa62 suggest the absence of these evasion mechanisms without compromising their growth.

RWC indicates coffee water status, showing reduced plants subjected to water stress and reflecting the balance between water uptake and transpiration [74,86]. Notably, plants inoculated with *Pantoea* sp. RCa62, while showing a lower leaf RWC compared to those under full irrigation conditions, do not differ statistically from the plants without stress. In contrast, their RWC is higher than that of control plants and several other groups under similar stress conditions despite their higher evapotranspiration and leaf area.

To date, we have not found other studies exploring the effect of PGPR inoculation on coffee under drought conditions regarding RWC and other functional attributes. However, the impact of an ACC deaminase-producing strain of *Pseudomonas* sp. and *Serratia marcescens* on improving the water status of wheat plants, evaluated through RWC, has been highlighted [87]. Similar results were found in plants derived from sterilized seeds inoculated with *Bacillus subtilis* Rhizo SF 48, also an ACC deaminase-producing strain [88].

### 3.4. Bacterial Effect on Plant Growth

In this study, regarding the parameters of primary growth, it is noteworthy that although significant differences were not observed concerning uninoculated controls in well-irrigated plants, the inoculation effect becomes clear under drought conditions. Considering that evapotranspiration can influence photosynthetic rate and plant growth, plants’ absolute and relative primary growth inoculated with *Pantoea* sp. RCa62 under water deficit conditions stands out and is comparable to plants under higher soil volumetric water content (VWC). This could be associated with higher evapotranspiration and leaf area than plants in other drought groups. Irrigation regimes had a notable effect on primary and secondary growth, as one of the main effects of water stress is reduced plant growth, and inoculation with growth-promoting rhizobacteria has proven to be a viable strategy to mitigate crop productivity losses in this and other studies [22,24,89]. Previous studies have evaluated the effect of *Kocuria* sp., *Bacillus subtilis*, *Sagenomella diversispora*, and *Penicillium ochrochloron*, all phosphate-solubilizing species, on the growth of coffee seedlings from sterilized seeds. These plants showed more significant growth compared to the non-inoculated controls [90]. However, their response under water stress conditions was not assessed. In this study, all plants in the cluster with the highest growth were inoculated with phosphate-solubilizing strains (*Serratia* RCa28, *Pantoea* RCa31, and RCa62).

Although no effects of the treatments were observed on total biomass, a significant irrigation effect was identified on dry mass allocation, with root development being prioritized in stressed plants compared to those fully irrigated. It is well established that drought drastically alters resource allocation, as prioritizing root growth enables water and nutrient uptake even under water scarcity conditions [86,91,92]. In this study, plants associated with *Pantoea* sp. RCa62 had the heaviest roots, likely facilitating the growth of larger shoots. Moreover, those inoculated with this strain showed better leaf bud production among the stressed plants, generating significantly more new foliar tissue than other stressed groups, including uninoculated controls. We propose that enhanced root development supports and enables the growth of the aerial parts of the plants. In this regard, the group of fully irrigated plants associated with RCa31 and RCa28 and stressed plants in symbiosis with RCa62 exhibited more developed roots and an intermediate root-to-shoot ratio. A well-developed root system supports shoot growth, which, in turn, can meet the plant’s metabolic needs and maintain productivity [92,93].

### 3.5. Bacterial Effect on Root Development

Root architecture plays a significant role in plant resistance to drought in addition to root weight or biomass partitioning [92,94,95]. In this study, it was shown that the proportion in length of taproot is 25% greater in plants without water restrictions since more complex root architectures in stressed plants, reflected as a lower mean root diameter but longer roots, allow a better water and nutrient uptake efficiency under water scarcity conditions [95]. Even more, the longest roots were found in plants under drought and inoculated with RCa62, the bacterial strain with the most significant IAA production. Among the plant growth regulators, auxins play a substantial role in promoting root elongation, and the effect of these phytohormones depends upon their overall concentration [96,97,98]. Many studies have reported the impact of IAA-producing microorganisms on drought stress resistance through root elongation in some plants such as maize, chickpea, pepper, *Vigna radiata*, wheat, alfalfa, and white clover [21,24,99,100,101,102]. Regarding cuttings on Robusta coffee, IAA-producing *Bacillus wiedmannii* and *Rhodococcus qingshengii* effectively increased the number of primary roots [103]. This study does not explore the effects on plants subjected to water restrictions.

Moreover, ACC deaminase activity enhances the effect of IAA through cross-talk communication between ethylene and IAA [104]. While IAA activates plant ACC synthase transcription, leading to increased ethylene levels that inhibit IAA signal transduction, bacterial ACC deaminase reduces ethylene levels in the plant [29]. This reduction also diminishes the feedback inhibition on IAA signaling. Consequently, in the presence of ACC deaminase, bacterial IAA can continue to promote plant growth and increase ACC synthase transcription with minimal ethylene feedback inhibition. Thus, the synergy between IAA and ACC deaminase lowers plant ethylene levels, allowing IAA to stimulate plant growth more effectively [28].

Therefore, a larger photosynthetic surface, which is more susceptible to water loss through transpiration via the stomata, along with the consequent increased evapotranspiration, and together with the absence of detrimental effects on RWC and growth parameters, suggests a mechanism that enhances water uptake. Given that the drought regime was moderate and prolonged, the success of the plants associated with RCa62 may be attributed to the expansion of the root system. Additionally, it is essential to mention that although the evapotranspiration of plants treated with *Pantoea* sp. RCa62 is higher than that of most drought-affected groups, it remains significantly lower than that of plants without water restrictions, indicating a possible greater water use efficiency (WUE, i.e., the amount of biomass produced per unit of water consumed), a phenomenon observed in other cases of plants with limited water availability [105]. It has previously been reported that the association of crops with PGPR can increase their WUE under water deficit conditions due to various plant growth promotion mechanisms [106,107].

It is worth noting that *Stenotrophomonas* sp. RCa07, *Sphingobacterium* sp. RCa08, *Raoultella* sp. RCa12, *Chryseobacterium* sp. RCa13, *Pantoea* sp. RCa37, and *Pseudomonas* sp. RCa18 demonstrated less favorable performance in one or more parameters related to primary growth, secondary growth, shoot production, leaf area, and relative water content under drought conditions. Therefore, at least in similar situations, they are not recommended as potential bioinoculants.

### 3.6. Bacterial Effect on Pigment Concentrations

Contrary to what has been documented in other studies conducted on wheat and maize [108,109], the amount of chlorophylls was not sensitive to the plant’s water conditions. However, other research on diverse coffee cultivars determined that total chlorophyll contents only decline until drought stress is intensified, and the decrease is less evident in drought-resistant cultivars [110]. Given that the present study was performed under moderate drought and with a drought-resistant cultivar such as Costa Rica 95, the absence of response in this parameter is explained. On the other hand, other studies have found that the ratio of chlorophyll a/b is a parameter that has not been modified in plants subjected to water stress [109]. Like chlorophyll, the total carotenoid concentration was not different among the groups. However, the carotenoids/chlorophyll ratio differed between the non-inoculated groups under high water availability and drought conditions and when comparing all plants from the irrigation treatments (Supplementary Table S2). Carotenoids are responsible for dissipating excess energy in the leaves and removing free radicals such as singlet oxygen, with an increased carotenoids/chlorophyll ratio potentially serving as a tolerance mechanism under stress conditions, mainly due to the photoprotective effect required in drought-stressed plants [111]. This effect was not promoted by any of the strains used in this research but by the irrigation regimes.

### 3.7. Bacterial Effect on Proline Accumulation

**Figure 8:**
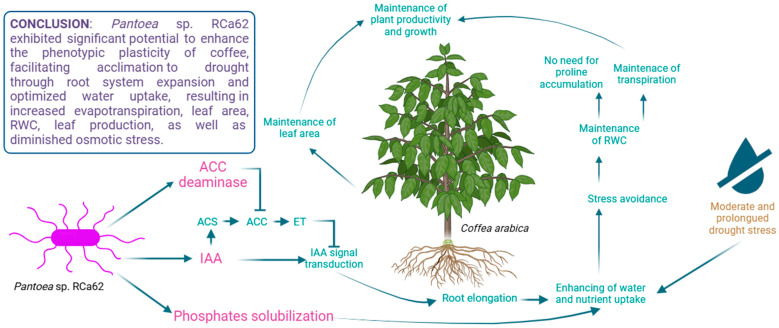
*Pantoea* sp. RCa62, a phosphate-solubilizing bacterium that produces IAA and ACC deaminase, promotes root elongation and water and nutrient uptake in young *Coffea arabica* plants through these synergistic mechanisms. Root development is also notably enhanced under drought stress. These modifications in root architecture serve as a stress avoidance strategy and help maintain morphophysiological parameters that support plant growth and productivity. Bacterial secretions or processes are highlighted in pink, while their effects on the plants are highlighted in green. Arrowheads indicate positive relationships between mechanisms, while barred arrows indicate inhibitory relationships. (ACC: 1-aminocyclopropane-1-carboxylic acid; ACS: ACC synthase; ET: ethylene; IAA: indole-3-acetic acid; RWC: relative water content). Created in BioRender (https://www.biorender.com/, accessed on 27 February 2023).

Proline is a crucial osmolyte that accumulates under osmotic stress and promotes plant growth as a tolerance strategy [112,113]. In well-irrigated control plants and those inoculated with *Pantoea* sp. RCa62 under both irrigation regimes, proline levels were significantly lower than in non-inoculated drought-stressed plants. The maintenance of lower proline levels in plants associated with *Pantoea* sp. RCa62, even under drought conditions, indicates low osmotic stress in the leaf tissue. This is likely due to improved water acquisition efficiency from enhanced root system development, which supports drought resistance through avoidance mechanisms rather than tolerance [114,115]. However, plant responses to drought stress may combine both strategies [93]. These findings are consistent with those of Bhise and Dandge [116], who observed reduced proline accumulation in rice plants inoculated with *Pantoea agglomerans* KL, even under salt stress, suggesting that this symbiosis mitigates stress effects. The results also align with the general trend of increased proline accumulation as water status declines [117], as seen also in coffee plants [118]. The integration of all the effects of *Pantoea* sp. RCa62 on coffee morphophysiology under moderate and prolonged drought conditions is shown in Figure 8.

### 3.8. Further Remarks

Plant growth promotion traits evaluated in vitro do not necessarily imply success in vivo implementation under controlled conditions, and even less so in field conditions. That is the case with strains of the *Pantoea* genus (RCa31, RCa37, RCa62), whose plant growth-promoting features were similar, but the results obtained in in vivo assays with non-sterile soil varied drastically. RCa31 showed promising results in promoting plant growth under well-irrigated conditions, RCa62 performed well under water scarcity, and RCa37 showed poor results in primary and secondary growth parameters. The same case is evident in *Pseudomonas* sp. RCa18, which displayed the highest ACC deaminase activity and other interesting growth-promoting traits but did not show the expected effects on coffee shrubs. Variations in the microbial strain performance under in vitro conditions compared to non-sterile soils can be explained by several factors: interactions with other microorganisms, nutrient competition, and pathogen presence influence their effectiveness [119]. Environmental conditions such as water availability or soil temperature may affect the outcomes; for example, RCa62 could be better adapted to water stress, while RCa31 is more effective with adequate irrigation. Additionally, strains’ action mechanisms, such as hormone production and nutrient solubilization, vary in effectiveness depending on soil context, making growth promotion potential under controlled conditions not always extendible to optimal field performance [37,120].

Moreover, the efficiency of bioinoculants can vary depending on factors such as root exudate composition, presence of organic matter, nutrient availability, organic acids, metals, and phytohormones in the soil [121,122]; conducting experiments closer to field conditions is essential. This approach helps to elucidate the complex biochemical, ecological, and evolutionary factors influencing the bacteria-plant-soil system and leads to more effective biotechnological applications. Consequently, results obtained from experiments using sterile seeds and substrates are necessary but insufficient for accurately determining a strain’s potential as an effective bioinoculant. There is a need for information derived from extensive evaluations under real-world conditions to fully understand the potential of microbial strains to promote plant growth in different environmental contexts [37,121].

In the same way, coffee displays different resistance strategies depending on the severity of stress [25]. In this case, root development was a pivotal trait promoted by *Pantoea* sp. RCa62, allowing water uptake under progressive and moderate drought conditions, thus enabling the implementation of morphological responses such as leaf area modification. However, coffee’s strategies may vary under more severe drought regimes and repeated drought events [9]. Further assessment of this strain in adult shrubs is also needed, as the bioinoculant effects may extend to yield and quality outcomes.

In this study, coffee phenotypic plasticity is enhanced by *Pantoea* sp. RCa62, which improves drought resistance and makes this strain a promising potential bioinoculant for reducing the vulnerability of coffee plantations to water scarcity predicted as a consequence of climate change.

## 4. Materials and Methods

### 4.1. Sampling

In December 2021, samples of the rhizosphere of *Coffea arabica* L. var. Costa Rica 95 were collected at the experimental site Teocelo-INIFAP (Instituto Nacional de Investigaciones Forestales, Agrícolas y Pecuarias). INIFAP is in Teocelo, Veracruz, Mexico (19°23′33.6″ N, 97°00′03.5″ W) at 1300 m above sea level, with an average annual precipitation of 1972 mm and a mean annual temperature of 18.91 °C. These climatic conditions are considered optimal for coffee cultivation [123].

Nine points were selected along three zigzag lines covering the entire plot for sample collection. Soil was extracted from each point at a diameter of 60 cm around the coffee shrub trunks, extending to the four cardinal points at 0–15 cm depth, where it has been previously documented that most of the coffee roots are found, and the influence of coffee roots is highly evident due to water and soil uptake [124,125,126]. The soil samples were placed in sealable bags, transported, and stored at room temperature in a dark environment, avoiding excessive heat.

The experimental plot includes eight shade tree species arranged in a randomized block design with three replicates for each species. The tree species present in the agronomic trial are *Roseodendron donnell-smithii* (Rose) Miranda, *Juglans pyriformis* Liebm., *Pinus chiapensis* (Martínez) Andresen, *Grevillea robusta* A. Cunn. ex R. Br., *Inga vera* Willd., *Cordia alliodora* (Ruiz and Pav.) Oken, *Tapirira mexicana* Marchand, and *Tabebuia rosea* (Bertol.) DC. The soil is described as sandy loam. No pest or weed control practices are implemented. Coffee shrubs receive annual fertilization with 200 g of a 4:2:2 mixture of urea, potassium chloride (KCl), and diammonium phosphate (DAP 18-46-0).

### 4.2. Isolation and Selection of ACC Deaminase-Producing Rhizobacteria

The collected soil used for isolation was embedded in the root network, so the roots were shaken to remove the attached soil [127]. No sonication or washing protocols were carried out in order to avoid excessive loss of rhizosphere soil, as the influence of plant root presence extends to the soil up to 2 cm from the root surface [128]. To select isolates with the capacity for ACCd production, 1 g of soil (two replicates per sample) was added to vials containing 20 mL of Dworkin and Foster (DF) liquid saline medium [129] with ACC as the sole nitrogen source at a final concentration of 3 mM. The tubes were incubated at 30 °C and 160 rpm for 48 h [55]. Subsequently, subculturing was performed by taking 1 mL of the culture and inoculating it into 19 mL of sterile DF + ACC medium, followed by incubation under the same conditions. Next, DF + ACC medium plates were inoculated. To prepare a solid medium, 30 µmol of ACC per plate was added as the sole nitrogen source and dispersed using sterile glass beads. An amount of 20 µL of the previously cultured DF + ACC broth was inoculated and spread by cross-streaking. Various colonial morphologies on the plates were identified and subcultured onto LB plates until axenic cultures were obtained to isolate microorganisms. A total of 54 purified isolates were obtained and stored at −70 °C in 35% glycerol. The isolates were kept in the collection of the Microbial Biotechnology Laboratory at ENCB-IPN.

A bacterial growth assay was performed in microplates to confirm ACCd production and select the isolates with the highest growth, using only ACC as the nitrogen source. Cryopreserved bacteria were subcultured onto LB plates for 48 h at 30 °C, then inoculated into 5 mL of LB broth and incubated for 24 h at room temperature. The LB cultures were washed twice with 10 mM MgSO_4_ before inoculation. Microplate wells were prepared with 122 µL of DF medium, 15 µL of a solution containing various nitrogen sources (ACC 0.032 M as the treatment, MgSO_4_ 0.1 M as the negative control, and (NH_4_)_2_SO_4_ 0.1 M as the positive control), along with 22 µL of inoculum. Isolates were inoculated in triplicate. Additionally, triplicate wells without inoculum were prepared for each condition, with 22 µL of MgSO_4_ 10 mM instead of bacterial culture [55,130]. Microplates were incubated at room temperature for 72 h with shaking at 150 rpm. Optical density (OD) readings at 600 nm were obtained using a Multiskan FC microplate reader (Thermo Scientific, Woodlands, Singapore). To account for OD contributions from reagents, the mean OD of wells without inoculum was subtracted from the readings of each condition. OD600 values were analyzed using ANOVA to identify isolates with higher growth in DF + ACC compared to the MgSO_4_ negative control (post-hoc Tukey, *p* < 0.05).

Isolates that exhibited growth in media with ACC as the only nitrogen source were taxonomically identified using primers 27F (5′-GTGCTGCAGAGACTTTGATCCTGGCTCAG-3′) and 1492R (5′-CACGGATCCTADGGGTACCTTACGACT-3′) to amplify the 16S rRNA gene sequence. The PCR products were sequenced using Sanger sequencing at Macrogen (Seoul, Republic of Korea). The sequences were edited and assembled using Chromas Pro (Technelysium), and the consensus sequences were analyzed with EzBioCloud (https://www.ezbiocloud.net/ (accessed on 27 February 2023) for identification. BOX-PCR analyzed the strains of the same genera for clonal identification using the primer BOXA1R (5′-CTACGGCAAGGCGACGCTGACG-3′) [131]. All rhizobacteria 16S rRNA gene sequences were deposited to NCBI GenBank with accession numbers (PQ449695–PQ449706).

To confirm the identity of the genera of the strains belonging to the order Enterobacteriales (RCa01, RCa28, RCa12, RCa31, RCa37, and RCa2), a phylogenetic analysis was performed based on 16S gene sequences. The analysis incorporated the sequences of the available type strains from EzBioCloud (https://www.ezbiocloud.net/ (accessed on 21 January 2023) that showed high similarity with the study sequences (similarity > 98%). A multiple alignment was performed using MUSCLE (3.8) [132] to conduct a maximum likelihood analysis with IQ-TREE multicore version 1.6.1 [133], using the K2P + I + G4 nucleotide substitution model, based on the BIC values.

### 4.3. Characterization of Plant Growth-Promoting Traits on ACC Deaminase-Producing Strains

The activity of ACCd was determined based on the induction of ACCd production by the strains and measuring the production of α-ketobutyrate from ACC [55]. Statistical differences in ACCd activity were analyzed using the Kruskal-Wallis test (post-hoc Conover-Iman). Siderophore production and phosphate solubilization were determined by inoculating 2 μL of a culture adjusted to a density of approximately 6 × 10^8^ CFU/mL in quadruplicate onto agar with Chrome Azurol S (CAS medium) [134] and NBRIP medium [135], respectively. Plates were incubated at 30 °C for eight days. Measurements of halo diameter corresponding to iron solubilization in CAS medium were calculated as the total halo diameter minus the colony diameter. The phosphate solubilization index (*PSI*) in NBRIP medium was determined by the formula PSI=total diameter/colony diameter [34]. Statistical differences in iron and phosphate solubilization were analyzed using ANOVA (post-hoc Tukey) among the isolates with activity.

Indole-3-acetic acid production was evaluated in Jain and Patriquin medium [136] using the colorimetric method with Salkowski reagent [137]. For this, 100 μL of a culture adjusted to a density of approximately 6 × 10^8^ CFU/mL was inoculated into media both with and without tryptophan to assess the synthesis of this metabolite via tryptophan-dependent and independent pathways at 72 h. All measurements were performed in triplicate and read using a LAMBDA XLS spectrophotometer (Perkin Elmer, Waltham, MA, USA) at 540 nm. Significant differences among IAA-producing isolates were identified using Kruskal-Wallis (post-hoc Conover-Iman) tests.

Similarly, the strains were assessed for nitrogen fixation using the acetylene reduction assay [138,139] in BMGM medium [140]. Gas chromatography analyzed them with a Clarus 580 system (Perkin Elmer, Waltham, Massachusetts, USA) equipped with a flame ionization detector.

### 4.4. Pot Experiment Design

Ten-month-old *C. arabica* cv. Costa Rica 95 plants were purchased from a certified nursery in La Estanzuela, Emiliano Zapata, Veracruz. They were transplanted into a substrate composed of 60% commercial black soil (Nutrigarden, Tierra Negra, 30 kg bag), 20% coconut fiber, and 20% perlite. Pest management included using color traps, concentrated neem, and garlic commercial extract (BioNutra, Neem All) at a concentration of 10 mL/L of water and manual removal of ectoparasites. Plants were maintained in a 6-week acclimation period before inoculation. The plants were irrigated during this period to keep the soil moisture close to field capacity (85% VWC). The soil’s volumetric water content (VWC) was monitored using a Yieryi LY-201 soil moisture sensor (Shen Zhen Yage Technology Co., Ltd., Shenzhen, China).

After the acclimation period, each bacterial strain was inoculated in groups of 14 plants. The inoculum was prepared in 100 mL of LB broth in a 250 mL flask, shaking orbitally at 120 rpm at room temperature for 18 h. Bacteria were recovered by centrifugation and resuspended in 0.01 M MgSO_4_ to achieve a concentration of approximately 10^8^ CFU/mL. A total of 8 mL of the solution was inoculated per plant weekly into the rhizospheric soil for five weeks [101].

After completing the inoculation treatment, each group was divided into two subgroups: one received irrigation to maintain soil moisture near field capacity (R, 85% VWC). Meanwhile, the other subset (S) experienced a two-phase reduction in humidity. During the first phase, soil moisture was kept at 70% VWC for three weeks, and in the second phase, it was lowered to 55% VWC for five weeks. Based on the plants’ needs, irrigation was carried out every 48 to 72 h. This way, groups of seven plants were inoculated with each strain, along with a non-inoculated control group, under moderate and prolonged drought conditions (S) and regular irrigation (R). Evapotranspiration for each plant was recorded gravimetrically between irrigations, and the average was calculated every 48 h.

This experiment was conducted in the greenhouse of the Centro de Estudios Científicos y Tecnológicos “Miguel Othón de Mendizábal”, Instituto Politécnico Nacional. The light and temperature conditions were not directly controlled; ventilation measures and external shading techniques, such as leaf litter, were used to reduce extreme temperatures and simulate light incidence in an agroforestry system. All the plants were distributed uniformly and randomly across the greenhouse area to avoid biases caused by peak irradiance times or minor differences in the microclimate.

### 4.5. Effects of ACC Deaminase-Producing Strains on the Growth and Physiological Status of Coffea arabica

At the end of each treatment, the functional leaf attributes were measured: the leaf area and specific leaf area (SLA) were observed using ImageJ 1.54d [141]. Relative water content (RWC) was determined gravimetrically [142,143]. Leaf discs with a diameter of 1.5 cm were hydrated by floating them in water at approximately 20 °C for 3 to 4 h, avoiding darkness and maintaining consistent illumination. After hydration, the discs were quickly blotted dry with filter paper and weighed to obtain the turgid weight. They were then dried in an oven at 80 °C for 48 h, cooled in a desiccator, and weighed again to determine the dry weight. Leaf functional attributes were measured between 8.00 and 10.00 AM.

Additionally, the following growth parameters were assessed: height; absolute and relative primary growth; diameter at 5 cm from the ground; absolute and relative secondary growth; number, fresh weight, and dry weight of leaf buds; shoot and root biomass production; and root architecture, with the latter analyzed using RhizoVision Explorer v2.0.2 [144]. Chlorophyll a, b, and carotenoid concentrations were determined using the method of Hiscox and Israelstam [145], which involves fractionating 100 mg of leaf tissue into uniform pieces of 1 to 4 mm^2^ in 10 mL of dimethyl sulfoxide (DMSO) and extracting the pigments without homogenizing the tissue at 65 °C for 4 h. Absorbances at 665, 649, and 480 nm were measured to determine pigment concentrations using the formulas according to previously standardized formulas [146]. Young and fully expanded leaves from the upper third of the plant, specifically between the second and third pair of branches, were used for all leaf tissue tests, ensuring a clean cut.

### 4.6. Proline Accumulation

Proline content was determined in uninoculated plants and those associated with *Pantoea* sp. RCa62 using a colorimetric method with ninhydrin [147].

### 4.7. Data Analysis

All statistical analyses were conducted using R version 4.3.2. To group the strains according to the previously determined plant growth-promoting traits, a k-means analysis was performed using the heatmaps library version 1.0.12. The obtained values for each characteristic were normalized to a maximum value of 1. Multivariate analyses associated with plant traits in response to inoculation and drought treatments were performed using libraries factoextra version 1.0.7 and NbClust version 3.0.1. To determine the sources of variability and detect differences between groups, ANOVA tests were performed over plant parameters after verifying the assumptions of each test and transforming the data when necessary. Means were compared using Duncan’s test as well as Dunnett’s. All the plots were built with ggplot2 version 4.4.4.

## 5. Conclusions

Climate change underscores the need to develop agricultural systems adaptation strategies, particularly for vulnerable crops like coffee. This study primarily focused on exploring ACC deaminase-producing rhizobacteria in coffee plantations. Twelve ACC deaminase-producing bacterial strains were identified and characterized from the rhizosphere of *Coffea arabica* L. cv. Costa Rica 95, all belonging to Gram-negative genera known for their plant growth-promoting abilities under stress. Notably, *Pantoea* sp. RCa62, RCa31, and *Serratia* sp. RCa28 demonstrated the ability to enhance plant growth and drought resistance (in the case of RCa62) and promote growth under adequate irrigation (for the latter strains). This study highlights the variability in bioinoculant performance between in vitro and in vivo assays, emphasizing the need for extensive evaluations under real-world conditions to fully assess their effectiveness. Specifically, *Pantoea* sp. RCa62 exhibited significant potential to enhance the phenotypic plasticity of coffee, facilitating drought acclimation through root system expansion and optimized water uptake. This resulted in increased evapotranspiration, leaf area, relative water content (RWC), leaf production, and reduced osmotic stress. These findings suggest that *Pantoea* sp. RCa62 is a promising candidate for bioinoculant formulation aimed at reducing the vulnerability of coffee plantations to water scarcity, which is projected to worsen due to climate change. It is still necessary to evaluate the strain’s performance in coffee plants in productive fields and explore the possibility of optimizing inoculation by evaluating various methods and potential matrices to prolong the inoculum’s presence in the soil. Similarly, it is interesting to explore the strain’s mechanisms of action through the modification of gene expression associated with drought resistance in plants.

## Figures and Tables

**Table 1:** ACCd-producing strains and characterization of plant-growth-promoting attributes.

Strain	ACCd (μmol α-Ketobutyrate/mg Protein/h)	Nitrogen fixation	Iron Solubilization by Siderophores (Halo Diameter in mm)	PSI	IAA Production at 72 h (μg/mL)
*Serratia* sp. RCa01	1.13 ± 0.31 ef	–	7.0 ± 0.8 a	1.6 ± 0.1 e	1.36 ± 0.25 c
*Serratia* sp. RCa28	0.65 ± 0.14 fg	–	6.8 ± 1.0 ab	1.4 ± 0.1 e	–
*Stenotrophomonas* sp. RCa07	1.83 ± 0.09 de	–	–	–	Not determined
*Sphingobacterium* sp. RCa08	0.38 ± 0.14 g	–	–	–	–
*Sphingobacterium* sp. RCa20	0.37 ± 0.03 g	–	–	–	–
*Raoultella* sp. RCa12	1.22 ± 0.26 ef	–	6.0 ± 0.0 ab	1.7 ± 0.2 de	–
*Chryseobacterium* sp. RCa13	2.93 ± 0.08 cd	–	–	–	Not determined
*Pantoea* sp. RCa31	9.32 ± 0.42 a	–	–	2.7 ± 0.3 ab	27.73 ± 2.32 ab
*Pantoea* sp. RCa37	2.76 ± 0.27 cd	–	–	3.2 ± 0.2 a	25.26 ± 1.30 b
*Pantoea* sp. RCa62	5.77 ± 0.15 ab	–	–	2.3 ± 0.3 bc	30.23 ± 1.19 a
*Pseudomonas* sp. RCa18	9.90 ± 1.07 a	–	6.5 ± 0.6 ab	1.6 ± 0.2 e	–
*Pseudomonas* sp. RCa58	4.66 ± 0.55 bc	–	5.5 ± 0.6 b	2.1 ± 0.1 cd	–

Data represent the mean of at least three replicates ± SD. Three replicates were used for ACCd activity and IAA production, and four replicates were used for siderophore production and PSI. Values with different letters significantly differ at *p* = 0.05 (ACCd and IAA: Kruskal-Wallis test, post-hoc Conover Iman; PSI and siderophores: ANOVA, post-hoc Tukey). ACCd, ACC deaminase; PSI, phosphate solubilization index; IAA, indole-3-acetic acid.

**Table 2:** Effect of ACC deaminase-producing rhizobacteria on primary and secondary growth of *Coffea arabica* L. cv. Costa Rica 95 after eight weeks of soil humidity regimes near field capacity (R, 85% VWC) and under drought conditions (S, 55% VWC).

Strain	Primary Growth (cm)		Relative Primary Growth (%)		Secondary Growth (cm)		Relative Secondary Growth (%)	
	R	S	R	S	R	S	R	S
Control	2.4 ± 0.3 abcde	0.9 ± 0.3 fgh	6.16 ± 1.38 abcd	2.26 ± 0.62 fg	0.05 ± 0.03 ab	0.01 ± 0.04 abcdefg	7.08 ± 4.32 ab	1.55 ± 6.39 abcdef
*Serratia* sp. RCa01	2.5 ± 0.9 abcd	1.0 ± 1.2 efgh	6.04 ± 2.10 abcde	2.59 ± 3.05 efg	0.03 ± 0.02 abcde	0.05 ± 0.08 a	4.38 ± 3.16 abcde	8.61 ± 13.85 a
*Serratia* sp. RCa28	3.1 ± 0.8 ab	1.7 ± 1.1 bcdefg	7.26 ± 2.27 ab	4.11 ± 2.53 bcdefg	0.03 ± 0.0 abc	0.01 ± 0.03 abcdefg	4.83 ± 4.23 abcd	1.73 ± 4.33 abcdef
*Stenotrophomonas* sp. RCa07	2.4 ± 1.8 abcd	1.2 ± 0.8 defgh	6.09 ± 4.37 abcd	3.20 ± 2.10 defg	0.04 ± 0.03 abc	−0.01 ± 0.05 cdefg	5.94 ± 4.59 abc	−1.06 ± 7.17 cdef
*Sphingobacterium* sp. RCa08	2.2 ± 0.9 abcdef	1.3 ± 0.5 defgh	5.56 ± 1.97 abcdef	3.50 ± 1.93 cdefg	0.02 ± 0.03 abcdefg	0.01 ± 0.03 abcdefg	3.35 ± 4.4 abcdef	1.62 ± 4.61 abcdef
*Sphingobacterium* sp. RCa20	1.8 ± 1.1 bcdefg	1.6 ± 1.4 cdefgh	4.54 ± 3.05 bcdefg	3.99 ± 3.26 bcdefg	0.03 ± 0.02 abcd	−0.02 ± 0.03 defg	4.52 ± 3.22 abcde	−3.18 ± 6.21 ef
*Raoultella* sp. RCa12	2.2 ± 1.7 abcdef	0.7 ± 1.4 gh	5.66 ± 4.35 abcdef	1.64 ± 3.30 g	0.03 ± 0.03 abc	−0.02 ± 0.05 efg	5.82 ± 4.51 abc	−2.51 ± 8.15 def
*Chryseobacterium* sp. RCa13	1.4 ± 0.8 cdefgh	0.7 ± 0.6 gh	3.50 ± 1.84 cdefg	2.03 ± 1.73 g	0.04 ± 0.04 ab	−0.02 ± 0.03 fg	6.75 ± 5.9 abc	−3 ± 4.76 def
*Pantoea* sp. RCa31	2.7 ± 0.5 abc	0.5 ± 0.6 gh	6.24 ± 1.21 abcd	1.59 ± 2.10 g	0.02 ± 0.01 abcdefg	0.02 ± 0.02 abcdef	3 ± 2.31 abcdef	3.72 ± 2.45 abcdef
*Pantoea* sp. RCa37	2.2 ± 0.3 abcdef	0.4 ± 1.4 h	5.79 ± 1.09 abcde	1.26 ± 3.62 g	0.02 ± 0.03 abcdefg	0 ± 0.03 bcdefg	4.28 ± 7.44 abcdef	0.19 ± 6.04 bcdef
*Pantoea* sp. RCa62	2.6 ± 0.6 abc	3.1 ± 0.8 ab **	6.84 ± 2.14 abc	7.19 ± 1.12 ab **	0.05 ± 0.03 ab	0.01 ± 0.01 abcdefg	7.69 ± 5.8 ab	1.24 ± 2.16 abcdef
*Pseudomonas* sp. RCa18	2.7 ± 0.7 abc	1.4 ± 0.8 cdefgh	7.20 ± 1.66 ab	3.39 ± 2.05 cdefg	0.03 ± 0.03 abcde	−0.03 ± 0.03 g	4.15 ± 4.62 abcdef	−3.57 ± 4.75 f
*Pseudomonas* sp. RCa58	3.3 ± 1.0 a	2.1 ± 0.8 abcdef	8.74 ± 2.84 a	5.70 ± 2.98 abcdef	0.03 ± 0.02 abcdef	0.03 ± 0.03 abcdef	4.22 ± 2.71 abcdef	4.05 ± 4.58 abcdef
Total mean	2.4 ± 0.23 A	1.3 ± 0.25 B	6.12 ± 0.59 A	3.26 ± 0.63 B	0.03 ± 0.01 A	0.004 ± 0.01 B	5.08 ± 1.01 A	0.72 ± 1.53 B

Values are presented as the mean ± 95% CI (n = 7). Different letters indicate significant differences between groups (*p* < 0.05; post-hoc Duncan). The lowercase letters represent comparisons between the mean values of all irrigation and inoculation treatments. In contrast, the uppercase letters indicate comparisons of the total means of each irrigation treatment (R or S). Asterisks represent significant differences compared to the control of each group (**: significant at 0.01; post-hoc Dunnett).

**Table 3:** Effect of ACC deaminase-producing rhizobacteria after eight weeks of soil humidity regimes on biomass accumulation and partitioning of *C. arabica* L. cv. Costa Rica 95 under full irrigation (R, 85% VWC) and under drought conditions (S, 55% VWC).

Strain	Dry Weight of the Shoot (g)		Dry Weight of the Root (g)		Total Dry Weight (g)	
	R	S	R	S	R	S
Control	10.4 ± 2.84 bcd	8.93 ± 1.95 d	5.29 ± 1.93 b	6.89 ± 2.29 b	15.68 ± 4.65	15.83 ± 4.04
*Serratia* sp. RCa01	9.68 ± 2.57 bcd	10.76 ± 3.37 abcd	5.01 ± 1.65 b	6.77 ± 3.34 b	14.69 ± 4.08	17.54 ± 6.61
*Serratia* sp. RCa28	13.18 ± 3.26 abc	11.98 ± 6.3 abcd	7.65 ± 2.82 ab	7.36 ± 3.87 b	20.83 ± 6.02	19.33 ± 10.16
*Stenotrophomonas* sp. RCa07	10.93 ± 3.3 abcd	11.11 ± 1.73 abcd	5.75 ± 2.5 b	6.83 ± 0.58 b	16.67 ± 5.57	17.94 ± 1.86
*Sphingobacterium* sp. RCa08	11.17 ± 2.33 abcd	9.02 ± 1.39 cd	6.52 ± 1.93 b	6.65 ± 1.77 b	17.69 ± 4.15	15.67 ± 3.12
*Sphingobacterium* sp. RCa20	10.9 ± 3.33 abcd	10.75 ± 2.56 abcd	5.58 ± 2.39 b	6.27 ± 2.23 b	16.47 ± 5.44	17.02 ± 4.57
*Raoultella* sp. RCa12	9.9 ± 1.87 bcd	8.71 ± 1.65 d	6 ± 2.17 b	6.2 ± 1.57 b	15.91 ± 3.98	14.91 ± 3.01
*Chryseobacterium* sp. RCa13	10.51 ± 2.73 abcd	8.97 ± 2.13 d	5.47 ± 1.47 b	5.2 ± 1.89 b	15.98 ± 3.54	14.17 ± 3.46
*Pantoea* sp. RCa31	13.75 ± 3.95 ab	10.19 ± 2.39 bcd	6.8 ± 2.56 b	5.76 ± 1.56 b	20.55 ± 5.95	15.95 ± 3.71
*Pantoea* sp. RCa37	9.95 ± 3.06 bcd	8.45 ± 1.68 d	4.68 ± 2.13 b	6.32 ± 2.13 b	14.63 ± 5.06	14.77 ± 3.63
*Pantoea* sp. RCa62	11.14 ± 2.47 abcd	14.9 ± 3.92 a **	5.6 ± 1.56 b	10.57 ± 3.25 a *	16.75 ± 3.32	25.48 ± 7.02
*Pseudomonas* sp. RCa18	10.02 ± 4.01 bcd	11.15 ± 2.47 abcd	6.05 ± 4.01 b	7.04 ± 1.05 b	16.07 ± 7.93	18.19 ± 3.21
*Pseudomonas* sp. RCa58	11.04 ± 1.88 abcd	10.66 ± 3.03 abcd	5.49 ± 1.77 b	5.95 ± 1.65 b	16.52 ± 3.13	16.62 ± 4.56
Total mean	10.97 ± 0.67	10.43 ± 0.71	5.84 ± 0.51 B	6.76 ± 0.54 A	16.80 ± 1.12	17.19 ± 1.20

Values are presented as the mean ± 95% CI (n = 7). Different letters indicate significant differences between groups (*p* < 0.05; post-hoc Duncan). The lowercase letters represent comparisons between the mean values of all the irrigation and inoculation treatments. In contrast, the uppercase letters indicate comparisons of the total means of each irrigation treatment (R or S). Asterisks represent significant differences compared to the control of each group (*: significant at 0.05, **: significant at 0.01; post-hoc Dunnett).

**Table 4:** Effect of ACC deaminase-producing rhizobacteria after eight weeks of soil humidity regimes on bud production of *C. arabica* L. cv. Costa Rica 95 under full irrigation (R, 85% VWC) and drought conditions (S, 55% VWC).

Strain	Number of Leaf Buds		Fresh Weight of the Leaf Buds (g)		Dry Weight of the Leaf Buds (g)	
	R	S	R	S	R	S
Control	3.9 ± 1.6 abcd	2.3 ± 2.4 bcd	1.54 ± 1.32 abcd	0.25 ± 0.38 g	0.40 ± 0.33 abcd	0.08 ± 0.11 f
*Serratia* sp. RCa01	3.7 ± 1.8 abcd	3.0 ± 2.1 bcd	1.41 ± 0.59 abcde	0.90 ± 0.66 bcdefg	0.39 ± 0.17 abcd	0.27 ± 0.21 bcdef
*Serratia* sp. RCa28	4.1 ± 1.9 abcd	4.6 ± 3.6 abc	1.57 ± 0.57 abc	0.85 ± 0.58 bcdefg	0.35 ± 0.16 abcde	0.29 ± 0.21 bcdef
*Stenotrophomonas* sp. RCa07	2.7 ± 1.1 bcd	2.1 ± 2.0 cd	1.05 ± 0.57 bcdefg	0.45 ± 0.52 fg	0.27 ± 0.16 bcdef	0.15 ± 0.18 def
*Sphingobacterium* sp. RCa08	3.0 ± 1.2 bcd	2.4 ± 1.7 abcd	0.95 ± 0.24 bcdefg	0.55 ± 0.54 efg	0.26 ± 0.08 bcdef	0.18 ± 0.16 def
*Sphingobacterium* sp. RCa20	3.0 ± 1.3 bcd	2.4 ± 1.5 bcd	1.41 ± 1.04 abcde	0.34 ± 0.36 g	0.37 ± 0.29 abcd	0.10 ± 0.11 ef
*Raoultella* sp. RCa12	4.0 ± 1.8 abcd	2.9 ± 0.9 bcd	1.40 ± 0.89 abcde	0.51 ± 0.36 efg	0.35 ± 0.22 abcde	0.17 ± 0.13 def
*Chryseobacterium* sp. RCa13	3.9 ± 1.3 abcd	3.1 ± 2.2 bcd	1.15 ± 0.55 bcdefg	0.64 ± 0.49 cdefg	0.30 ± 0.14 bcdef	0.19 ± 0.14 cdef
*Pantoea* sp. RCa31	5.9 ± 2.0 a	2.4 ± 1.9 bcd	2.17 ± 1.51 a	0.64 ± 0.56 cdefg	0.57 ± 0.38 a	0.19 ± 0.16 cdef
*Pantoea* sp. RCa37	4.0 ± 1.6 abcd	2.0 ± 1.2 d	1.36 ± 0.86 abcdef	0.28 ± 0.20 g	0.36 ± 0.18 abcde	0.09 ± 0.07 f
*Pantoea* sp. RCa62	3.7 ± 1.3 abcd	4.7 ± 3.3 ab	1.72 ± 0.87 ab	1.57 ± 1.15 abc ***	0.45 ± 0.19 ab	0.45 ± 0.33 ab **
*Pseudomonas* sp. RCa18	3.0 ± 1.4 bcd	2.3 ± 1.8 bcd	0.90 ± 0.57 bcdefg	0.36 ± 0.37 g	0.24 ± 0.16 bcdef	0.11 ± 0.11 ef
*Pseudomonas* sp. RCa58	3.9 ± 0.7 abcd	2.6 ± 2.2 bcd	1.68 ± 0.78 ab	0.63 ± 0.70 defg	0.44 ± 0.22 abc	0.18 ± 0.19 def
Total mean	3.8 ± 0.3 A	2.8 ± 0.5 B	1.41 ± 0.18 A	0.61 ± 0.13 B	0.37 ± 0.05 A	0.19 ± 0.04 B

Values are presented as the mean ± 95% CI (n = 7). Different letters indicate significant differences between groups (*p* < 0.05; post-hoc Duncan). The lowercase letters represent comparisons between the mean values of all the irrigation and inoculation treatments. In contrast, the uppercase letters indicate comparisons of the total means of each irrigation treatment (R or S). Asterisks represent significant differences compared to the control of each group (**: significant at 0.01, ***: significant at 0.001; post-hoc Dunnett).

**Table 5:** Effect of ACC deaminase-producing rhizobacteria after eight weeks of soil humidity regimes on root development of *C. arabica* L. cv. Costa Rica 95 under full irrigation (R, 85% VWC) and drought conditions (S, 55% VWC).

Strain	Total Root Length (m)		Mean Root Diameter (mm)		Total Root Surface (cm^2^)		Total Root Volume (cm^3^)		Dry Shoot-to-Root Ratio	Total Root Length (m)
	R	S	R	S	R	S	R	S	R	S
Control	66.68 ± 37.68 b	100.65 ± 43.84 ab	0.66 ± 0.06 abc	0.64 ± 0.05 bc	1315 ± 629 b	1936 ± 775 ab	73 ± 34	86 ± 17	2.05 ± 0.33 ab	1.39 ± 0.38 f
*Serratia* sp. RCa01	55.32 ± 33.87 b	101.05 ± 45.29 ab	0.68 ± 0.07 abc	0.63 ± 0.08 c	1119 ± 588 b	1924 ± 826 ab	55 ± 20	81 ± 30	2.02 ± 0.32 ab	1.73 ± 0.35 abcdef
*Serratia* sp. RCa28	114.81 ± 70.23 ab	81.19 ± 33.65 ab	0.66 ± 0.08 abc	0.69 ± 0.04 abc	2164 ± 1152 ab	1706 ± 737 ab	100 ±37	78 ± 26	1.81 ± 0.27 abcde	1.63 ± 0.19 abcdef
*Stenotrophomonas* sp. RCa07	70.45 ± 40.07 b	84.18 ± 23.76 ab	0.68 ± 0.07 abc	0.68 ± 0.05 abc	1414 ± 743 b	1748 ± 383 ab	69 ± 33	81 ± 8	2.16 ± 0.68 ab	1.64 ± 0.28 abcdef
*Sphingobacterium* sp. RCa08	106.61 ± 51.81 ab	90.68 ± 28.29 ab	0.65 ± 0.11 bc	0.65 ± 0.05 abc	1972 ± 892 ab	1792 ± 503 ab	87 ± 35	75 ± 10	1.86 ± 0.5 abcde	1.44 ± 0.32 def
*Sphingobacterium* sp. RCa20	69.04 ± 51.97 b	75.76 ± 36.21 ab	0.71 ± 0.09 abc	0.69 ± 0.08 abc	1375 ± 895 b	1531 ± 588 ab	72 ± 38	71 ± 14	2.09 ± 0.4 ab	1.82 ± 0.44 abcde
*Raoultella* sp. RCa12	82.67 ± 41.45 ab	80.77 ± 17.37 ab	0.65 ± 0.05 abc	0.64 ± 0.03 bc	1624 ± 736 ab	1633 ± 334 ab	72 ± 34	74 ± 10	1.78 ± 0.36 abcdef	1.45 ± 0.33 cdef
*Chryseobacterium* sp. RCa13	53.34 ± 32.82 b	65.23 ± 28.73 b	0.77 ± 0.12 a	0.68 ± 0.04 abc	1116 ± 538 b	1342 ± 569 b	66 ± 17	62 ± 21	2.00 ± 0.47 abc	1.98 ± 0.87 abcdef
*Pantoea* sp. RCa31	98.77 ± 61.18 ab	70.1 ± 25.61 ab	0.63 ± 0.06 c	0.66 ± 0.02 abc	1869 ± 1020 ab	1423 ± 505 b	90 ± 44	65 ± 20	2.19 ± 0.75 ab	1.82 ± 0.33 abcde
*Pantoea* sp. RCa37	56.36 ± 42.95 b	92.92 ± 30.04 ab	0.74 ± 0.12 abc	0.63 ± 0.08 c	1162 ± 805 b	1774 ± 580 ab	58 ± 25	71 ± 27	2.37 ± 0.59 a	1.41 ± 0.3 ef
*Pantoea* sp. RCa62	74.42 ± 41.2 ab	138.89 ± 59.93 a	0.66 ± 0.07 abc	0.65 ± 0.06 bc	1412 ± 659 b	2684 ± 971 a	64 ± 23	109 ± 33	2.15 ± 0.65 ab	1.45 ± 0.24 cdef
*Pseudomonas* sp. RCa18	77.97 ± 83.26 b	81.04 ± 12.67 ab	0.75 ± 0.12 ab	0.63 ± 0.05 c	1477 ± 1416 b	1589 ± 177 ab	76 ± 59	68 ± 8	1.94 ± 0.5 abcd	1.59 ± 0.29 bcdef
*Pseudomonas* sp. RCa58	85.48 ± 59.29 ab	83.88 ± 31.86 ab	0.64 ± 0.06 bc	0.64 ± 0.06 bc	1600 ± 937 ab	1615 ± 462 ab	76 ± 37	75 ± 19	2.15 ± 0.52 ab	1.81 ± 0.26 abcde
Total mean	77.84 ± 11.56 B	88.18 ± 8.09 A	0.68 ± 0.02 A	0.65 ± 0.01 B	1509.22± 195.86 B	1745.98 ± 143.63 A	73.63 ± 7.79	76.40 ± 4.88	2.04 ± 0.11 A	1.63 ± 0.09 B

Values are presented as the mean ± 95% CI (n = 7). Different lowercase letters indicate significant differences between groups (*p* < 0.05; post-hoc Duncan). Distinct uppercase letters show differences between total means of irrigation treatments (*p* < 0.05).

## Data Availability

All the rhizobacteria 16S rRNA gene sequences were deposited to NCBI GenBank with accession numbers (PQ449695–PQ449706).

## Supplementary Materials

The following supporting information can be downloaded at: https://www.mdpi.com/article/10.3390/plants14071084/s1, Figure S1: Maximum-likelihood tree based on 16S rRNA gene sequences of the Enterobacteriales strains. Figure S2: Hierarchical clustering heatmap of the strains according to the relative values for each characterized plant growth-promoting trait; Table S1: Mean values and ANOVA for each plant parameter determined; Table S2: Pigment concentrations.

## Abbreviations

The following abbreviations are used in this manuscript:

ACC	1-aminocyclopropane-1-carboxylic acid
ACCd	ACC deaminase
PGPR	Plant growth promoting rhizobacteria
R	Full-irrigation treatment
RWC	Relative water content
S	Drought stress treatment
VWC	Volumetric water content
