# Supplementary material for: Beneficial Effects of ACC Deaminase-Producing Rhizobacteria on the Drought Stress Resistance of Coffea arabica L
Source: Plants (Basel). 2025 Apr 1;14(7):1084. doi: 10.3390/plants14071084 (PMC11991408; doi:10.3390/plants14071084)
Supplement: Supplementary file 1 [file plants-14-01084-s001.zip › plants-3512053-supplementary.pdf]

Supplementary materials

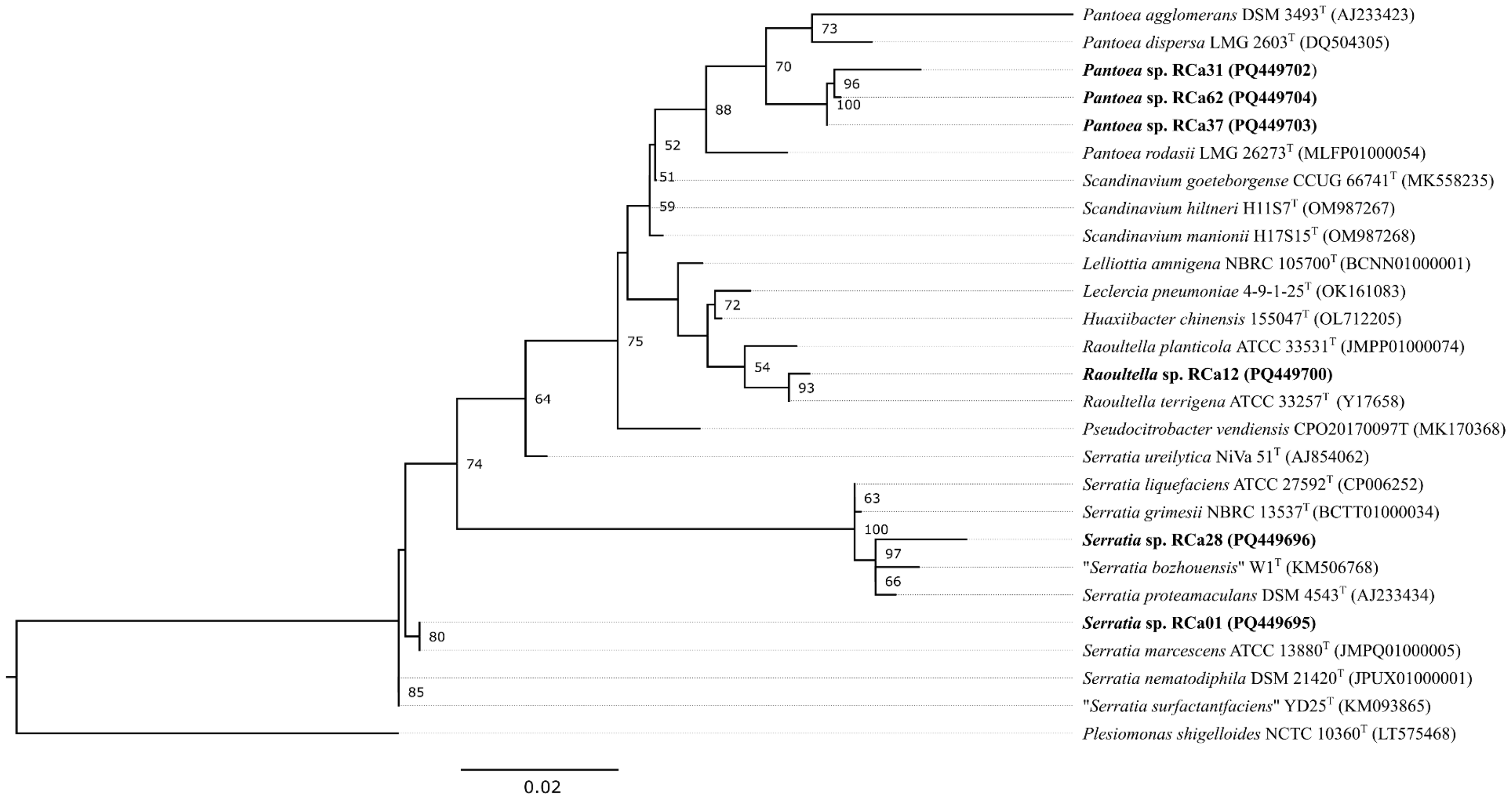

Figure S1. Maximum-likelihood tree based on 16S rRNA gene sequences of Enterobacteriales strains. Bootstrap values (1000 replicates) are shown as percentages rounded to the nearest integer ranging from 50 to 100. The sequence of *Plesiomonas shigelloides* NCTC 10360 was included as an outgroup. The scale bar represents the magnitude of 0.02 substitutions per site.

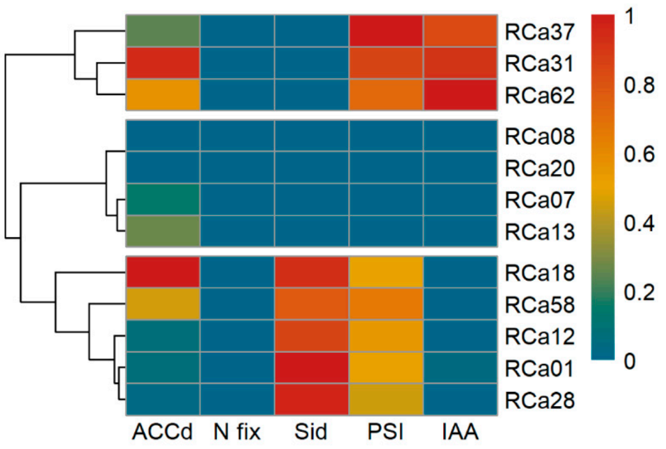

http://ijs.sgmjournals.org

Figure S2. Hierarchical clustering heatmap of the strains according to the relative values for each characterized trait. The values for each feature were normalized to a maximum of 1, and grouping was performed using k-means analysis.

Table S1. Mean values of plant parameters of each irrigation group and analysis of variance for plant metrics assessed in inoculation (In) and irrigation (Irr) treatments. ANOVA values are the mean squares of each variation source. (Df: degrees of freedom, *: significant at 0.05, **: significant at 0.01, ***: significant at 0.005).

	**Mean**		**ANOVA**						**Transform**
	**Full irrigation (85% soil VWC)**	**Drought (55% soil VWC)**	**Source**	**In**	**Irr**	**Irr*In**	**Residuals**	**Total**	
**Plant parameters**			**Df**	12	1	12	156	181	
Evapotranspiration over 48 h (mL)	175.74 ± 3.94	73.77 ± 2.77		361	473117***	281	259		
Leaf area	31.57 ± 1.60	25.02 ± 1.57		177.8***	1951.7***	54.7	48.8		
RWC	91.35 ± 0.65	87.07 ± 0.84		21.8***	1022.0***	9.0**	3.4		
SLA	210.20 ± 4.61	214.60 ± 4.35		209.5	879.6	214.5	501.8		
Initial height (cm)	40.0 ± 1.11	39.6 ± 1.04		30.569	8.835	17.837	27.021		
Final height (cm)	42.4 ± 1.15	40.9 ± 1.10		46.8·	113.14*	22.91	28.33		
Primary growth (cm)	2.4 ± 0.23	1.3 ± 0.25		4.01***	58.4***	1.58	1.09		
Relative primary growth (%)	6.12 ± 0.59	3.26 ± 0.63		23.9***	372.3***	8.2	7.4		
Initial diameter at 5 cm above the soil (cm)	0.65 ± 0.02	0.65 ± 0.02		0.008235	0.000198	0.004445	0.006365		

Final diameter at 5 cm above the soil (cm)	0.68 ± 0.02	0.65 ± 0.02		0.01019	0.03888*	0.00316	0.00684		
Secondary growth (cm)	0.03 ± 0.01	0.004 ± 0.01		0.00173	0.03517***	0.00243·	0.00139		
Relative secondary growth (%)	5.08 ± 1.01	0.72 ± 1.53		40.2	863.6***	60.0·	36.8		
Fresh weight of the shoot (g)	32.29 ± 2.06	30.13 ± 2.11		1.4622*	1.6610	0.7804	0.7189		sqrt
Fresh weight of the root (g)	22.92 ± 2.60	23.13 ± 2.10		149.56	1.93	155.46	25.42		
Total fresh weight (g)	55.21 ± 4.36	53.26 ± 3.99		612.0·	174.9	470.2	381.6		
Dry weight of the shoot (g)	10.97 ± 0.67	10.43 ± 0.71		20.37*	13.15	11.65	10.21		
Dry weight of the root (g)	5.84 ± 0.51	6.76 ± 0.54		0.2790	1.7064**	0.2643	0.2427		sqrt
Total dry weight (g)	16.80 ± 1.12	17.19 ± 1.20		0.6260	0.1126	0.4615	0.4174		sqrt
Number of leaf buds	3.75 ± 0.33	2.84 ± 0.46		5.54	37.85**	4.10	3.50		
Fresh weight of the leaf buds (g)	1.41 ± 0.18	0.61 ± 0.13		1.179*	28.858***	0.548	0.537		
Dry weight of the leaf buds (g)	0.37 ± 0.05	0.19 ± 0.04		0.0820*	1.4212***	0.0450	0.0394		
Total root lenght (m)	77.84 ± 11.57	88.18 ± 8.09		6.67	41.70*	7.50	7.22		sqrt
Mean root diameter (mm)	0.68 ± 0.02	0.65 ± 0.01		0.036	0.118*	0.038	0.028		-1/x
Total root surface (cm^2)	1509.22± 195.86	1745.98 ± 143.63		102.8	782.4**	113.5	108.2		sqrt
Total root volume (cm^3)	73.63 ± 7.79	76.40 ± 4.88		802.0	348.5	1286.4	962.9		
Fresh shoot-to-root ratio	1.66 ± 0.12	1.41 ± 0.09		0.3267	2.9640**	0.1915	0.2652		
Dry shoot-to-root ratio	2.04 ± 0.11	1.63 ± 0.09		0.0234	0.7305***	0.0216	0.0194		-1/x
Chlorophyll a concentration (μg/g fresh weight)	959.97 ± 82.65	960.50 ± 61.00		70702	13	145949	123689		
Chlorophyll b concentration (μg/g fresh weight)	377.71 ± 30.55	355.83 ± 27.35		9871	21766	23629	19790		
Total chlorophyll (μg/g fresh weight)	1337.68 ± 111.89	1316.33 ± 85.93		22.97	0.07	53	42.88		sqrt
Chl$_a$:Chl$_b$ ratio	2.56 ± 0.09	2.80 ± 0.10		0.1665	2.5555***	0.0721	0.2048		
Carotenoids (μg/g de fresh weight)	218.04 ± 16.05	225.04 ± 11.68		2445	2226	3534	4781		
Carotenoids:chlorophyll ratio	0.17 ± 0.006	0.18 ± 0.005		0.0024	0.0206*	0.0051	0.0038		log$_{10}$

Table S2. Effect of ACC deaminase-producing rhizobacteria on pigments concentrations on leaves of *Coffea arabica* cv. Costa Rica 95 after eight weeks of soil humidity regimes near field capacity (R, 85% VWC) and under drought conditions (S, 55% VWC).

	**Chlorophyll a (μg/g fresh weight)**		**Chlorophyll b (μg/g fresh weight)**		**Total chlorophyll (μg/g fresh weight)**		**Cl$_a$/Cl$_b$ ratio**		**Carotenoids (μg/g de fresh weight)**		**carotenoids/chlorophyll ratio**	
**Strain**	**R**	**S**	**R**	**S**	**R**	**S**	**R**	**S**	**R**	**S**	**R**	**S**

Control	1070 ± 300	806 ± 191	450 ± 88	292 ± 94	1520 ± 379	1098 ± 272	2.35 ± 0.34 c	2.86 ± 0.45 abc	233 ± 61	203 ± 40	0.15 ± 0.01 a	0.19 ± 0.02 b
Serratia sp. RCa01	888 ± 213	901 ± 145	349 ± 93	338 ± 71	1237 ± 301	1239 ± 203	2.59 ± 0.34 abc	2.75 ± 0.51 abc	211 ± 35	209 ± 33	0.17 ± 0.02 ab	0.17 ± 0.01 ab
Serratia sp. RCa28	978 ± 403	1047 ± 154	370 ± 163	349 ± 82	1348 ± 558	1396 ± 227	2.72 ± 0.55 abc	3.08 ± 0.43 a	236 ± 89	243 ± 24	0.18 ± 0.01 ab	0.18 ± 0.02 ab
Stenotrophomonas sp. RCa07	726 ± 177	998 ± 335	295 ± 83	363 ± 151	1021 ± 255	1361 ± 480	2.5 ± 0.31 abc	2.86 ± 0.3 abc	178 ± 31	235 ± 65	0.18 ± 0.02 ab	0.18 ± 0.02 ab
Sphingobacterium sp. RCa08	728 ± 271	1014 ± 346	290 ± 60	382 ± 121	1018 ± 327	1396 ± 461	2.44 ± 0.52 bc	2.65 ± 0.33 abc	191 ± 97	237 ± 69	0.18 ± 0.06 ab	0.17 ± 0.02 ab
Sphingobacterium sp. RCa20	928 ± 405	1088 ± 266	371 ± 122	410 ± 145	1299 ± 518	1498 ± 405	2.48 ± 0.46 bc	2.75 ± 0.37 abc	211 ± 91	251 ± 53	0.16 ± 0.02 ab	0.17 ± 0.02 ab
Raoultella sp. RCa12	998 ± 438	903 ± 453	379 ± 177	317 ± 182	1377 ± 612	1221 ± 633	2.73 ± 0.38 abc	2.98 ± 0.5 ab	227 ± 80	216 ± 77	0.17 ± 0.02 a	0.19 ± 0.03 ab
Chryseobacterium sp. RCa13	1137 ± 448	807 ± 246	428 ± 172	293 ± 101	1565 ± 617	1100 ± 343	2.69 ± 0.46 abc	2.84 ± 0.39 abc	234 ± 67	195 ± 45	0.16 ± 0.02 ab	0.18 ± 0.03 ab
Pantoea sp. RCa31	1012 ± 450	1032 ± 207	387 ± 175	430 ± 157	1399 ± 622	1462 ± 307	2.67 ± 0.32 abc	2.57 ± 0.57 abc	224 ± 79	226 ± 51	0.17 ± 0.03 ab	0.16 ± 0.03 ab
Pantoea sp. RCa37	1264 ± 444	948 ± 273	487 ± 140	341 ± 110	1751 ± 580	1289 ± 366	2.55 ± 0.36 abc	2.85 ± 0.46 abc	270 ± 88	230 ± 52	0.16 ± 0.01 ab	0.18 ± 0.02 ab
Pantoea sp. RCa62	801 ± 325	959 ± 283	331 ± 143	369 ± 108	1132 ± 464	1328 ± 390	2.48 ± 0.34 bc	2.61 ± 0.17 abc	185 ± 57	219 ± 51	0.17 ± 0.03 ab	0.17 ± 0.01 ab
Pseudomonas sp. RCa18	999 ± 389	994 ± 378	380 ± 113	370 ± 154	1380 ± 495	1364 ± 528	2.57 ± 0.48 abc	2.85 ± 0.56 abc	220 ± 75	235 ± 75	0.16 ± 0.01 ab	0.18 ± 0.03 ab
Pseudomonas sp. RCa58	952 ± 352	988 ± 147	393 ± 160	372 ± 81	1345 ± 510	1360 ± 224	2.5 ± 0.32 abc	2.71 ± 0.29 abc	215 ± 63	228 ± 28	0.17 ± 0.03 ab	0.17 ± 0.01 ab

	959.97 ± 82.65	960.50 ± 61.00	377.71 ± 30.55	355.83 ± 27.35	1337.68 ± 111.89	1316.33 ± 85.93	2.56 ± 0.09 B	2.80 ± 0.10 A	218.04 ± 16.05	225.04 ± 11.68	0.17 ± 0.006 B	0.18 ± 0.005 A

Values are presented as the mean ± 95% CI (n = 7). Different lowercase letters indicate significant differences between groups ($p < 0.05$; post-hoc Duncan). Asterisks represent significant differences compared to the control of each group (*: significant at 0.05, **: significant at 0.01, ***: significant at 0.001; post-hoc Dunnett). Distinct uppercase letters show differences between total means of irrigation treatments ($p < 0.05$).
